# Supplementary material for: Opioid distribution trends (2006–2017) in the US Territories
Source: PeerJ. 2019 Jan 15;7:e6272. doi: 10.7717/peerj.6272 (PMC6338096; doi:10.7717/peerj.6272)

| **Supplemental Table 1.** Demographics of three US territories, Florida and Hawaii. (American Fact Finder, 2018; Hernandez et al. 2017; Index Mundi, 2018; US Census, 2018). American Samoa had a 2017 population of 51,504, 88.9% native Samoans, 3.6% Asian, 1.2% other, and 0 Narcotic Treatment Programs. https://www.cia.gov/library/publications/the-world-factbook/geos/aq.html | | | | | |
| --- | --- | --- | --- | --- | --- |
|  | **Florida** | **Puerto Rico** | **Hawaii** | **Guam** | **US Virgin Islands** |
| Total population (2017) | 20,984,500 | 3,337,177 | 1,427,538 | 167,358 | 104,901 |
| White  Hispanic/Latino | 77.1%  22.5% | 78.6%  75.1% | 41.5% | 6.8% | 15.6% |
| Black/African American  Hispanic/Latino | 17%  0.8% | 14.8%  12.3% | 2.9% | *N/A | 76% |
| American Indian/Native Alaskan  Hispanic/Latino | 0.9%  0.1% | 0.3%  0.5% | 2.5% | 7.2% | *N/A |
| Asian  Hispanic/Latino | 3%  0.1% | 0.3%  0.1% | 57.4% | 32.5% | 1.4% |
| Native Hawaiian/Pacific Islander | 0.2% | 0.1% | 26.2% | 42.4% | *N/A |
| Other  Hispanic/Latino | 4.5%  3.4% | 9%  7.8% | 2.5% | 11.3% | 2.1% |
| Age  15-24 years  25-54 years  55 + years | 13.00%  39.80%  29.8% | 13.71%  38.10%  32.41% | 13.30%  40.90%  27.1% | 16.63%  37.44%  18.39% | 11.90%  37.07%  31.33% |
| Narcotic Treatment Programs (2017)^#^ | 57 | 6 | 4 | 0 | 1 |
| *N/A: not applicable, insufficient data due to small population size; ^#^Drug Enforcement Administration’s Automation of Reports and Consolidated Ordering System on 7/03/2018. | | | | | |

| **Supplemental Table 2.** Opioid comparison. | | | | |
| --- | --- | --- | --- | --- |
|  | **Brand/Street Name** | **Medical/Illicit Routes of Intake** | **Indications** | **Precautions** |
| **codeine^W-A^**  *(1/10-1/20 as potent as morphine)* | Captain Cody, Cody, Lean, Schoolboy, Sizzurp, Purple Drank, Loads, Pancakes and Syrup | oral, IV* | Typically found in cough medicine for antitussive effect |  |
| **fentanyl^W-A^**  *(An analgesic 100 times more potent than morphine*) | Actiq®, FentoraTM, Duragesic® | IV, transdermal patch, smoke, oral, snort | Often used as a potent analgesic and anesthetic. Oral transmucosal lozenges (fentanyl lollipops) are used for cancer pain management. Transdermal patches are used for chronic pain management. | Fentanyl is often abused for its intense euphoric effects and can serve as a more potent substitute for heroin. Overdoses can result from respiratory distress or lead to death. |
| **hydrocodone**  *(Equally as potent as morphine in treating pain)* | Vicodin®, Lortab®, Lorcet-HD®, Hycodan®, Vicoprofen® | oral , snorted, injected | Often this is used as a cough suppressant and to treat moderate/moderately severe pain. | Hydrocodone is often abused for its euphoric effects. It is very concerning for its prevalence in school-aged children (8th to 12th graders). High doses of acetaminophen-containing hydrocodone can produce liver toxicity over time. |
| **hydromorphone**  *(4x as potent as morphine)* | Dilaudid®, Dust, Juice, Smack, D, Footballs) | injected, rectal | Used to treat moderate to severe pain. | Overdoses can lead to severe respiratory depression, somnolence that progresses to a coma, skeletal muscle weakness, decreased blood pressure, and decreased heart rate. |
| **meperidine** |  | oral, snorted, injected |  |  |
| **methadone^W-A^**  *(3-5x as potent as morphine)* | Methadose®, Dolophine®, Fizzies, Amidone, Chocolate Chip Cookies | oral, injected | Primarily used to treat opioid addiction and for narcotic withdrawal. It is also used to treat moderate to severe pain. | Overdose of methadone could lead to severe respiratory distress, comas, pulmonary edema, reduced blood pressure, reduced heart rate, and death. Methadone abuse could lead to psychic and physiological dependence. |
| **morphine^W-A,W-C^** |  | Injected, oral, smoke |  |  |
| **oxycodone**  *(Equally as potent as morphine in relieving abstinence symptoms from chronic opiate use)* | Tylox®, Percodan®, OxyContin® | Oral, snorted, injected | Used for extended/ continuous treatment of moderate to severe pain. | Oxycodone is abused for its euphoric effects. |
| **oxymorphone**  *(3-7x as potent as morphine)* | Opana®, Opana ER®,  Blue Haven, Blues, Mrs. O, Octagons,Oranges, Pink Heaven, Pink lady, Stop Signs, The O Bomb. | Oral, snorted, injected | Used for treating moderate to severe pain. | Overdose of oxymorphone could lead to severe respiratory depression, coma, skeletal muscle flaccidity, reduced heart rate and blood pressure. Oxymorphone abuse can lead to circulatory collapse, cardiac arrest, and even death. |
| *IV: intravenous; ^W^World Health Organization 2017 list of essential medications for ^A^adults or ^C^children | | | | |

**Supplemental Figure 1.** Population for the US Territories. Guam is from US Census (2010), World Bank (<http://www.multpl.com/guam-population/table/by-year> for 2007-9, 2011-16), and Index Mundi ([https://www.indexmundi.com/guam/demographics_profile.html for 2017](https://www.indexmundi.com/guam/demographics_profile.html%20for%202017). Puerto Rico is from the US Census (2010) and American Community Survey (all other years). American Samoa and the US Virgin Islands are from the US Census (2010, 2017). ^e^estimated based on assumption of a linear rate of population change from 2000 to 2010 or from 2010 to 2017. Change in population from 2006 to 2017 are in parentheses.


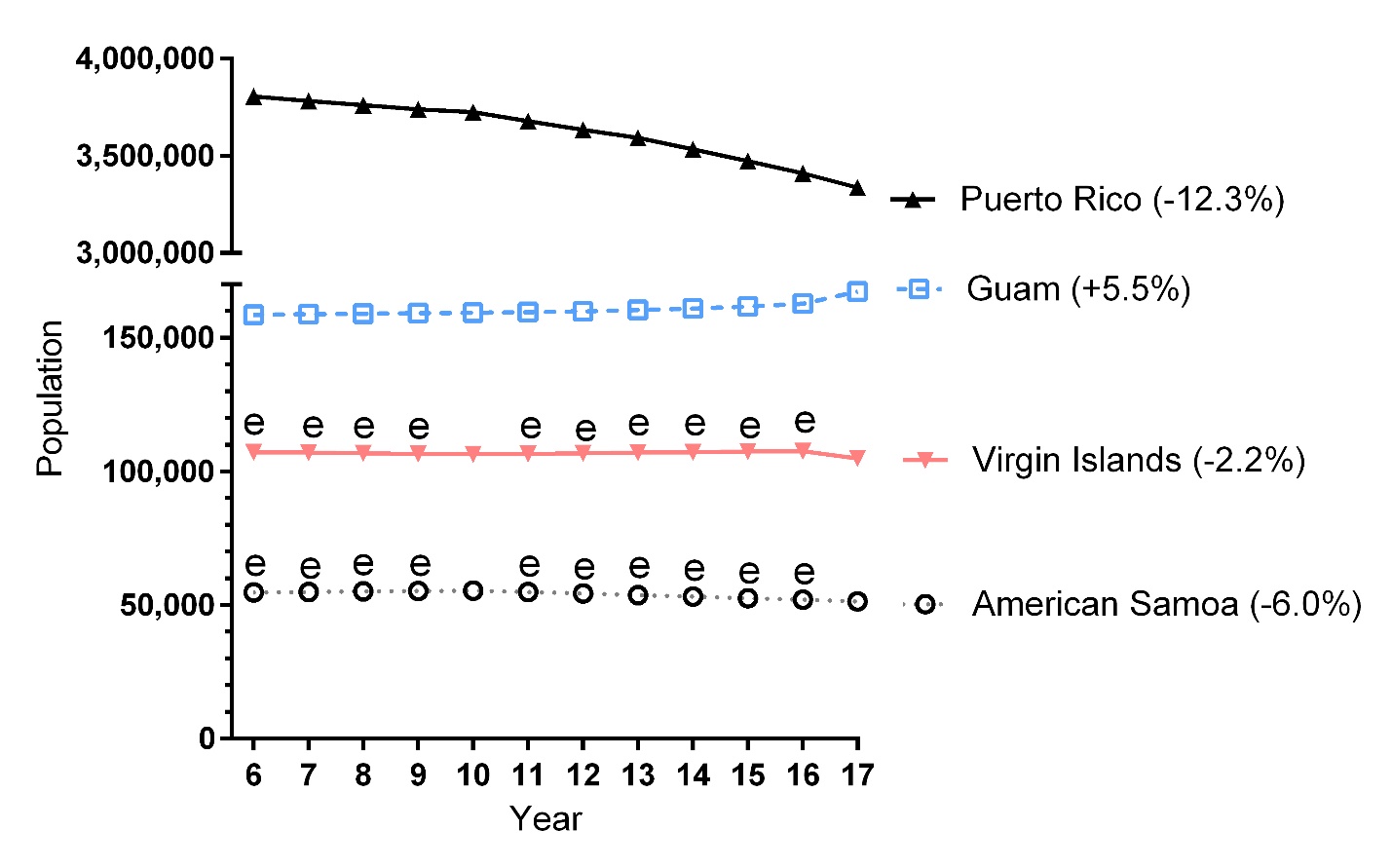


**Supplemental Figure 2.** Percent of the total morphine mg equivalent (MME) for ten opioids in 2017 as reported (Run Date = 7/03/2018) by the US Drug Enforcement Administration’s Automation of Reports and Consolidated Ordering System (ARCOS) for Puerto Rico (A), Florida (B), and all municalities covered by ARCOS (C).. Oral MME conversions: buprenorphine: 10; codeine: 0.15; fentanyl base: 75; hydrocodone: 1; hydromorphone: 4; meperidine: 0.1; methadone: 12 from Narcotic Treatment Programs (NTP), 8 from all other sources; morphine: 1; oxycodone: 1.5, and oxymorphone: 3. For methadone, total percentage and percentage from non-NTPs (pharmacy, hospital, practitioners/mid-level practitioners, teaching institution) are shown in parentheses.

**
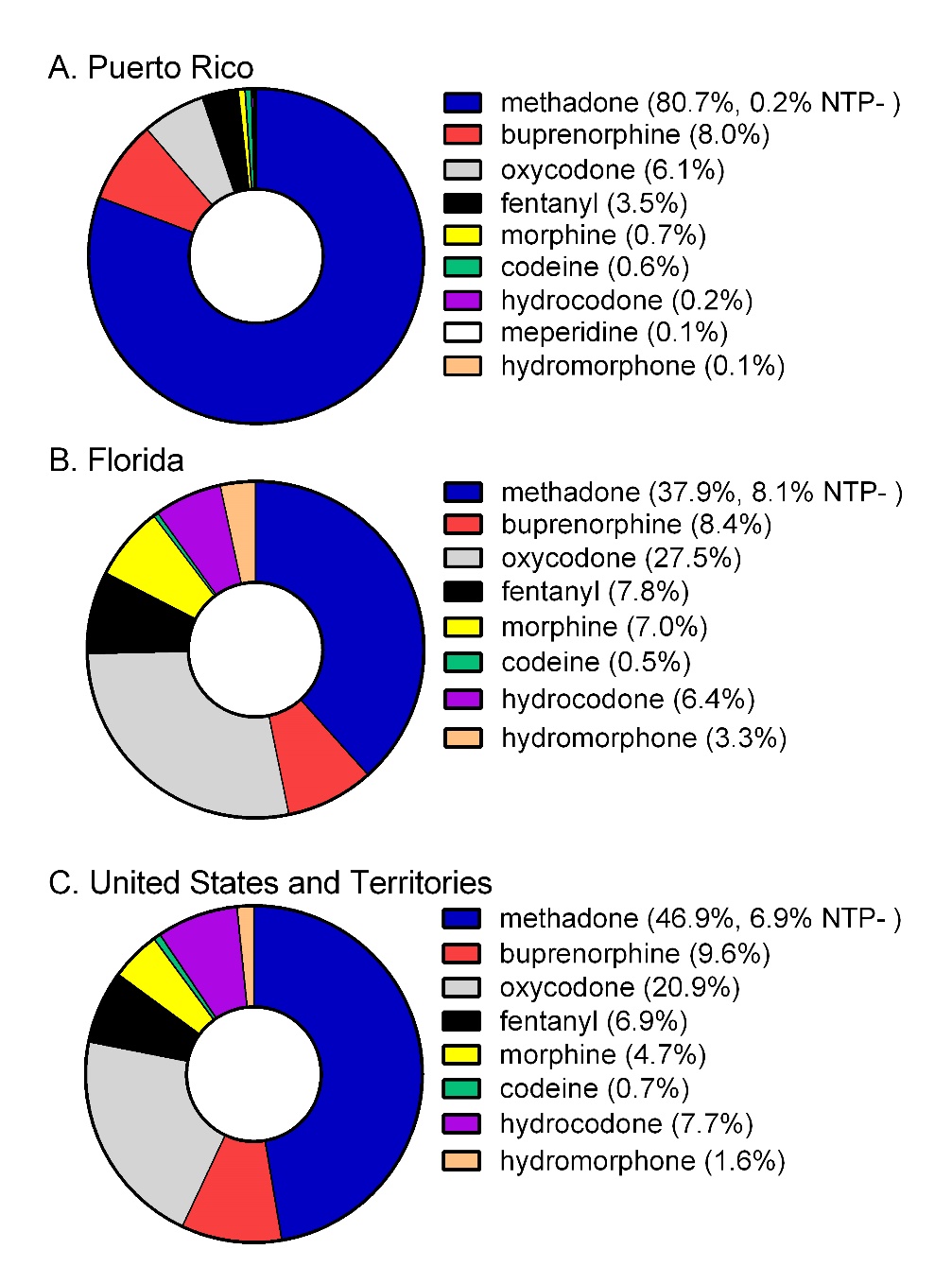
**

**Supplemental Figure 3**. Percent of the total oral morphine mg equivalent (MME) for ten opioids by business activity as reported by the US Drug Enforcement Administration’s Automation of Reports and Consolidated Ordering System for Puerto Rico (A), Florida (B), or all states including Washington DC and US Territories (C). Oral MME conversions: buprenorphine: 10; codeine: 0.15; fentanyl base: 75; hydrocodone: 1; hydromorphone: 4; meperidine: 0.1; methadone: 10; morphine: 1; oxycodone: 1.5, and oxymorphone: 3.


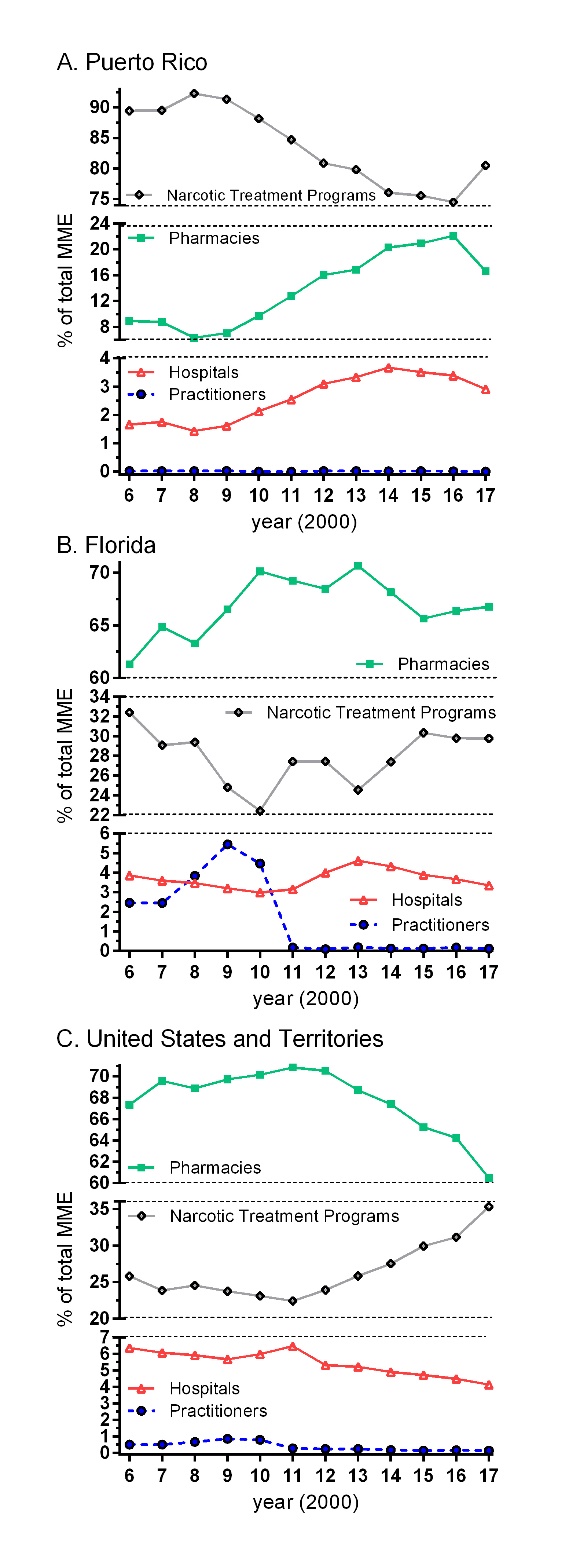

Supplement: Supplemental Information 1 — Sup Table 1. Demographics of three US territories, Florida and Hawaii. Sup Table 2. Opioid comparison. Sup Fig 1. Population for the US Territories. Sup Fig 2. Percent of the total morphine mg equivalent (MME) for ten opioids in 2017. Sup Fig 3. Percent of the total oral morphine mg equivalent (MME) for ten opioids by business activity. [file peerj-07-6272-s001.docx]
